# Supplementary material for: LEM-3/ANKLE1 nuclease prevents the formation of abnormal syncytium to safeguard neuronal differentiation
Source: PLoS Biol. 2026 Nov 2;24(11):e3004099. doi: 10.1371/journal.pbio.3004099 (PMC13743039; doi:10.1371/journal.pbio.3004099)
Supplement: Supplementary file 1 [file pbio.3004099.s015.docx]

Response to Reviewer Feedback for PBIOLOGY-D-26-00728R2

We thank all three reviewers for their constructive comments. A point-by-point response is provided below, with reviewers’ critiques in blue text and authors’ response in black text. We are also providing a “track-changes” style version of the manuscript that shows changes and edits.

Reviewers' comments

Reviewer 1:
This study identified a function for the C. elegans endonuclease LEM-3 in the separation of sister cells during the final cell division preceding terminal differentiation and fate determination. When LEM-3 is absent, chromatin bridges remain unresolved, leading to failed cytokinesis and the development of binucleate syncytia, evident by a "canal" remaining between sister cells. The failure to separate sister cells during cytokinesis impacts cell neuronal differentiation, resulting in sister cells in some cases acquiring the same cell fate. In some cases where the sibling cell fate is apoptosis, lem-3 mutants showed a loss of apoptosis. Studies of fluorescently labelled cell fate markers and cell fate determinants indicate that these proteins may diffuse in the canal. The results are mostly convincing, and the manuscript is well-written. However, although their study provides detailed explanations to the duplication of sister cell fate in cytokinesis mutants, one could argue that the findings are not surprising and do not in themselves point to a novel general principle in developmental biology.

Major issues
Figure 1: they refer to the connections between sister cell in lem3 mutants as "canals". But could instead be axon/dendrite projections between the cells. What is the difference between a "canal" and an aberrant axon/dendrite?

Response: As suggested by reviewer #2, we have replaced the term “intercellular canal” with “stable intercellular bridge” to better reflect the fact that they are the result of incomplete cytokinesis. Given the fact that the bridge only occurs between sister cells in genetic mutants that lacks the ability to resolve chromatin linkages, we do not think they originated from aberrant neurite outgrowth. However, like an aberrant neurite, the bridge contains microtubules and actin filaments, suggesting that they may be stabilized/maintained as a neurite. We have already mentioned this point in the manuscript.

Figure 2: I find the labelling in the figure confusing. Are all these images from a lem-3 mutant background? How do they score that there is a syncytium? According to Figure 1, the "canal" is only formed in ~20% of cases.

Response: Yes. All the images shown in Figure 2 are from *lem-3(-)* mutants. We first identified the syncytium based on the *mec-17p::GFP* marker, which labels the entire syncytium with a bridge connecting two cells. We then count the percentages of syncytia that shows smFISH signals in either AVM cell body only or in both SDQR and AVM. The images for wild-type animals are shown in Figure S5. We have revised the legends to clarify the issue.

Figure 3: The diffusion of proteins through the canal between the two sister cells is open for other interpretations. For instance, the presence of MEC3::GFP in both sister cells does not automatically mean that MEC3::GFP protein has diffused through the canal. Instead, the diffusion of other proteins may trigger the same cell fate in both sister cells and hence result in the expression of many cell fate markers. To definitively claim that MEC3::GFP and other proteins diffuse through the canal they would have to activate the reporter protein expressions at a later stage and study its diffusion.

Response: To further confirm that MEC-3 drives the TRN fate in the sister cells, we created *lem-3(-); mec-3(-)* double mutants and found that the sister cells no longer expressed the TRN fate marker and syncytium only expressed the sister cell fate markers. In the case of ALM and PLM, their sisters (BDU and ALN) were not transformed into the TRN fate upon the loss of *mec-3*, supporting that MEC-3 is required for the fate conversion observed in the binucleate syncytium in *lem-3(-)* mutants. These results are added to the new Figure S4 in the revised manuscript.

Minor issues
Introduction, 1st paragraph: The "final round of cell division…" is over-simplified. In general terms, when introducing the topic and referring to many biological settings, it can come in several very different forms: 1) both sister cells differentiating, and into different fates, 2) both sister cells differentiating, but into the same fate, or 3) one cell differentiating, often referred to as the "daughter cell", while the other, the "mother cell" retains some level of progenitor properties and continues dividing.

Response: We have now added more clarifications to the first paragraph in the revised manuscript and mentioned the three scenarios.

Should not unk14 and unk85 be unc-14 and unc-85?

Response: *“unk”* is the allele designation from our lab (the CGZ lab). Both *unk14* and *unk85* are *lem-3* alleles (Figure 1A) and do not refer to the *unc-14* and *unc-85* genes.

Reviewer 2:
The manuscript by Deng and Zheng reports the characterization neuronal fate specification in animals lacking the function of LEM-3, a midbody-localized nuclease. The authors uncovered an allele of lem-3 in a screen for extra touch receptor neurons (TRNs). They show that these mutants maintain a stable cytoplasmic bridge (which they call intercellular canal) between TRNs and their sister cells. The bridge contains actin, tubulin and DNA/chromatin and enables cytoplasmic exchange between sister cells. The authors demonstrate that the bridge correlates with a proportion of sister cells adopting a TRN fate, likely due to the diffusion of TRN-promoting factors, including MEC-3. The authors propose that the presence of chromatin bridges, which are typically processed by LEM-3, results in the long-term stabilization of the intercellular bridge in terminally-differentiating neurons and that this impacts neuronal fates by enabling the exchange of proteins between the two sister cells.

Overall I find the manuscript to be of excellent quality, with a sound experimental design and original findings that should be of interest to neurobiologists and cell biologists alike. The main finding that chromatin bridges can result in the stabilization of the intercellular bridge in cells undergoing terminal division prior to differentiation is novel, and the authors convincingly show that this bridge enables the sharing of cytoplasmic constituents, and thus can have an impact on cell fate. I have a few suggestions that the authors should consider to improve the quality of their work.

My main suggestion is in regards to lines 133-149, where the authors postulate that the fate change in TRN sister cells is due to diffusion of the TRN fate specifying factor MEC-3. They seek to test this idea by showing that ectopic expression of MEC-3 is sufficient to induce a fate change, however I do not think that this ectopic expression really tests their idea. I understand that part of the idea is that molecules can diffuse through the stable canal, including MEC-3 itself (as shown in Figure 3), however this does not show that MEC-3 is functionally competent. I think that the simplest way to show that the fate change is due to the presence of MEC-3 is to test whether the fate change phenotype is suppressed by the loss of MEC-3. Of course such gross perturbation will not demonstrate that it is the MEC-3 present in the TRN cell that induces the fate change in its sister, however it should unambiguously assess whether the presence of MEC-3 is needed for this fate change.
I think that this functional assessment is important given that the authors take for granted (in this and subsequent sections) that their fate change phenotype is caused by MEC-3 diffusion.

Response: We thank the reviewer for the suggestion. During the revision, we created *lem-3(-); mec-3(-)* double mutants and found that TRN-sister syncytia no longer expressed the TRN fate marker and only expressed the sister cell fate marker. In the case of ALM-BDU and PLM-ALN syncytium, the sister cells (BDU and ALN) were not converted into the TRN fate and still express their own fate markers. These results (added to Figure S4 in the revised manuscript) support that the diffusion of MEC-3 into the sister cells could cause cell fate transformation.

Minor suggestions and concerns
The authors refer to the connection as an intercellular canal, but this is a somewhat novel term that does not serve to anchor their work in biology. When cells divide they form a transient intercellular bridge around a midbody, and abscission leads to the severing of the bridge (membrane scission) and release of the midbody. In certain cells that undergo incomplete cytokinesis (e.g. germ cells), the intercellular bridge is stabilized, thus forming a stable intercellular bridge. I suggest that the authors employ the term stable intercellular bridge (or stable cytoplasmic bridge) instead of intercellular canal to better anchor their finding into the existing work on these structures.

Response: We agree with the reviewer that the term “canal” may not be informative in this scenario and have systematically changed “intercellular canal” to “intercellular bridge” in the revised manuscript.

I wonder whether the term syncytium is appropriate to refer to cells that remain connected by a stable bridge as a result of cytokinesis failure. My understanding of syncytia is that they can be generated by incomplete cytokinesis but this is a programed feature that is highly regulated, which is not the case here. They can also arise of pathological conditions (e.g. viral infections) but it is also different than what is observed here. I understand why the authors equate their cell pairs to syncytia but my opinion is that the term is not employed appropriately in this context. I suggest to refer to stably connected sister cells rather than syncytial cells.

Response: We think the idea of syncytium is important in our case because we want to emphasize the sharing of cytoplasmic content between the sister cells in *lem-3(-)* mutants. This sharing results in cell fate mixing or conversion, which are defects in terminal cell differentiation. In our opinions, the loss of *lem-3* causes a type of pathological binucleate syncytia where sister cells are abnormally connected. To make this idea clearer, we changed “syncytium” to “abnormal syncytium” in the title.

Lines 60-61: As far as I know, whether the entire C. elegans germline is formed as a result of incomplete cytokinesis is still an open question. Previous work has shown that the germline founder cell undergoes incomplete cytokinesis (PMID: 29074566) and that the first division of the PGCs may be incomplete (PMID: 34195824), however the notion that there is a sustained incomplete cytokinesis program when the germline expands remains hypothetical and some work suggests other mechanisms. The authors could instead write that "the germline of C. elegans hermaphrodites arises via incomplete cytokinesis and forms a syncytium in which more than 1000 nuclei share cytoplasm through stable bridges".

Response: We thank the reviewer for pointing out this issue and have corrected the sentence. We also added the citation to the two references mentioned above.

Line 70: define TRN (touch receptor neuron) in this sentence.

Response: We have now spelled out the “TRNs” in the first subheading of the Results.

Line 77, "…we mapped unk14 to the V618M mutation in lem-3…": Please rewrite. V618M is an amino acid change, not a mutation that can be mapped. The allele mapped to a change at the nucleotide level, which results in a V618M change in the resulting protein.

Response: We rewrote the sentence in the revised manuscript as “we mapped *unk14* to a mutation in *lem-3*, which codes for a midbody-tethered, LEM domain-containing DNA endonuclease. The mutation caused a V618M substitution in the LEM-3 protein.”

Lines 209-213 and Figure 3D: I find that the experiment to demonstrate that the stable canal is needed for cytoplasmic exchange is somewhat convoluted. The authors show that if they sever the canal they see a decrease in converted Dendra2 levels in the non-TNR sister, but this occurs over 48 hours. Have the authors considered performing the photoconversion in animals in which the canal has already been cut (i.e. essentially doing the same experiment as in 3B but after the canal is cut)? It seems like this would be more direct to demonstrate that the canal enables cytoplasmic exchange.

Response: Figure 3D is intended to show that after the severing of the bridge SDQR would lose the expression of TRN genes, because the TagRFP proteins made in AVM no longer diffused into SDQR.

In the revised manuscript, we conducted the experiment the reviewer suggested. We cut the bridge and then performed photoconversion of Dendra2 in the TRN (e.g., ALM and AVM) of the syncytia and did not observe the increase in red fluorescence in the sister cells (e.g., BDU and SDQR). The results are shown in Figure S7. This finding further confirms that the intercellular bridge enables cytoplasmic exchange.

Lines 378-381 and Figure 6I: The authors report that inhibiting ced-3 (which blocks apoptosis) results in an increase in the surviving sister cell in cell pairs where one usually dies. I think that it would rather be more informative if the authors documented whether the decrease in 3-cell body syncytia that they reported in older animals (Figure 6E) is suppressed in ced-3 mutants. If this were the case it would support their claim that apoptosis is delayed in 3-cell syncytia.

Response: We thank the reviewer for the suggestion. During the revision, we scored the penetrance of three-body syncytia and found that indeed the age-dependent decrease in three-body syncytia was suppressed in *lem-3(-); ced-3(-)* double mutants (new Figure 6E). The frequency of three-body syncytia is generally higher in *lem-3(-); ced-3(-)* double mutants compared to *lem-3(-)* single mutants.

Reviewer 3:
Deng et al. utilize C. elegans as an experimental system to investigate how the failed cytokinesis affect the specification of postmitotic neuron cells. They found that the loss of LEM-3 endonuclease, which is responsible for chromatin bridges resolution during cytokinesis, leads to binucleate syncytia connecting TRNs and sister cells through intercellular canal. These intercellular canals allow the diffusion of cytoplasmic proteins, while the transcriptomes of the linked cells can be independently regulated. They further investigated the functional consequences of these connections and found that the cytoplasmic linkages between sister cells resulted in suppression of neuronal fates and prevention of apoptotic fates. Finally, they showed that LEM-3 has a general role in cell fate specification in the nervous system by resolving the chromatin bridges.
In general, this is a very exciting study that provides insight into the consequence of compromised cytokinesis in cell fate differentiation. The experiments are overall well executed, the results are clearly presented.

Major points:
1. While previous studies have shown LEM-3 recruitment to the midbody in embryos, its localization should also be examined in the division that produces TRNs and their sister cells.

Response: During the revision, we constructed a transgenic animal expressing both mCherry:ZEN-4 and LEM-3::GFP in the Q cell lineage and indeed found the localization of LEM-3 to the midbody in the cell division that produces AVM and SDQR. The results are now shown in Figure 5A.

2. Fig 1E. The authors should discuss the potential reasons for the significant variation in syncytium penetrance among different TRN subtypes.

Response: We do not have good explanation for the different syncytium penetrance among different TRN subtypes. We suspect that the different lineage history, development timing, and anatomic positions (including the physical distance with the sister cells) may all contribute to the variation in penetrance. We added some potential reasons in the revised manuscript.

3. Fig. 3 shows that proteins, but not mRNAs, diffuse freely throughout the syncytium. Could it be that mRNAs are degraded in the canal or actively blocked by a selective barrier?

Response: We think the diffusion of mRNAs is generally limited. For example, in the smFISH staining against various cell fate markers in the wild-type animals, we observed signals mostly in the cell body and not in the neurites, suggesting that mRNAs do not diffuse into neurites. Following the same logic, we think mRNAs generally do not diffuse into the canal.

4. Fig. 4A The analysis should be complemented by a control experiment demonstrating syncytium formation in wild-type worms treated with the DNA topoisomerase II inhibitor or subjected to mcm-7 RNAi.

Response: We have done such control experiments and did not observe any syncytium in wild-type animals treated with *mcm-7* RNAi or ICRF-193. We added this information to the revised manuscript.

5. Fig 6E shows that although the three cells are connected by intercellular canals, only the third cell can be removed without triggering programmed cell death in the entire syncytium. Given that cytoplasmic proteins can diffuse throughout the syncytium, it would be interesting to investigate whether the other two cells could also be labeled by an apoptotic marker such as CED-1::GFP.

Response: We thank the reviewer for the suggestion. During the revision, we crossed the apoptotic marker *smIs34[ced-1p::ced-1::GFP]* with *lem-3(-); uIs115[mec-17p::TagRFP]* but did not find any labeling by CED-1::GFP on AVM or the sister SDQR in the three-body syncytia. Unfortunately, we were not able to capture the moment the third cell body is labeled by CED-1::GFP either, likely because it is transient process that could happen at any time over a three-day period. Therefore, we decided to not include these data in the revised manuscript.

Minor points:
1. The abbreviation "TRN" should be spelled out as "touch receptor neuron (TRN)" upon its first use in the manuscript.

Response: We have now spelled out the “TRNs” as “touch receptor neurons (TRNs)” at the beginning of the Results session.

2.line 122 Fig. 1B does not seem relevant to this specific point. The supporting data appears to be in Fig.S2 A-B only.

Response: We thank the reviewer for pointing out the mistake. We have now corrected the error in figure citation.

3. Please indicate how the "~20%" value was quantified and where the data is presented. It is not evident in the current Fig. 4E. Also, statistical analysis is required for the quantitative data in Fig 4E.

Response: There was a mistake in arranging the Figure panels. Fig. 4G is supposed to be Fig. 4E. We have now corrected the mistake. The percentage of AVM-SDQR intercellular bridge showing nuclear envelop connection (marked by LMN-1::GFP) is around 20% at 24-hour as indicated by the new Fig. 4E. For the new Fig. 4G (old Fig. 4E), we added a note in the figure legend to indicate that there is no statistically significant difference across the three time points.

4. Please add the figure citation (Fig. 5D) to the sentence on lines 307-311 regarding drug treatments.

Response: The figure citation is now added.
