# Supplementary material for: LEM-3/ANKLE1 nuclease prevents the formation of abnormal syncytium to safeguard neuronal differentiation
Source: PLoS Biol. 2026 Nov 2;24(11):e3004099. doi: 10.1371/journal.pbio.3004099 (PMC13743039; doi:10.1371/journal.pbio.3004099)
Supplement: Peer review history

# Editor report: Decision Letter 0

**DOI:** 10.1371/journal.pbio.3004099.r001

**Authors:** Ines Alvarez-Garcia

20 Mar 2026

Dear Dr Zheng,

Thank you for submitting your manuscript entitled "LEM-3/ANKLE1 nuclease prevents the formation of syncytium between postmitotic sister cells and safeguards neuronal differentiation" for consideration as a Research Article by PLOS Biology.

Your manuscript has now been evaluated by the PLOS Biology editorial staff as well as by an academic editor with relevant expertise and I am writing to let you know that we would like to send your submission out for external peer review.

However, before we can send your manuscript to reviewers, we need you to complete your submission by providing the metadata that is required for full assessment. To this end, please login to Editorial Manager where you will find the paper in the 'Submissions Needing Revisions' folder on your homepage. Please click 'Revise Submission' from the Action Links and complete all additional questions in the submission questionnaire.

Once your full submission is complete, your paper will undergo a series of checks in preparation for peer review. After your manuscript has passed the checks it will be sent out for review. To provide the metadata for your submission, please Login to Editorial Manager (https://www.editorialmanager.com/pbiology) within two working days, i.e. by Mar 24 2026 11:59PM.

If your manuscript has been previously peer-reviewed at another journal, PLOS Biology is willing to work with those reviews in order to avoid re-starting the process. Submission of the previous reviews is entirely optional and our ability to use them effectively will depend on the willingness of the previous journal to confirm the content of the reports and share the reviewer identities. Please note that we reserve the right to invite additional reviewers if we consider that additional/independent reviewers are needed, although we aim to avoid this as far as possible. In our experience, working with previous reviews does save time.

If you would like us to consider previous reviewer reports, please edit your cover letter to let us know and include the name of the journal where the work was previously considered and the manuscript ID it was given. In addition, please upload a response to the reviews as a 'Prior Peer Review' file type, which should include the reports in full and a point-by-point reply detailing how you have or plan to address the reviewers' concerns.

During the process of completing your manuscript submission, you will be invited to opt-in to posting your pre-review manuscript as a bioRxiv preprint. Visit http://journals.plos.org/plosbiology/s/preprints for full details. If you consent to posting your current manuscript as a preprint, please upload a single Preprint PDF.

Feel free to email us at plosbiology@plos.org if you have any queries relating to your submission.

Kind regards,

Ines

--

Ines Alvarez-Garcia, PhD

Senior Editor

PLOS Biology

ialvarez-garcia@plos.org

# Peer review: Decision Letter 1

**DOI:** 10.1371/journal.pbio.3004099.r002

**Authors:** Ines Alvarez-Garcia

28 Apr 2026

Dear Dr Zheng,

Thank you for your patience while your manuscript entitled "LEM-3/ANKLE1 nuclease prevents the formation of syncytium between postmitotic sister cells and safeguards neuronal differentiation" was peer-reviewed at PLOS Biology. The manuscript has now been evaluated by the PLOS Biology editors, an Academic Editor with relevant expertise, and by three independent reviewers.

The reviews are attached below. As you will see, the reviewers find the results interesting, but they also raise several issues that would need to be addressed before we can consider the manuscript for publication. Reviewer 1 raises several issues in the figures, including clarifications and an alternative interpretation of the observation on proteins diffused via the canal between the two sister cells. Reviewer 2 disagrees with the conclusion that the fate change in TRN sister cells is due to MEC-3 diffusion, and thinks that it should be shown that MEC-3 is functionally competent. In addition, this reviewer thinks it should be tested if the fate change phenotype is inhibited by the loss of MEC-3 to confirm that the presence of MEC-3 is needed for the fate change. Reviewer 3 mentions that the localisation of the LEM-3 recruitment to the midbody in embryos during the division that produces TRNs and their sister cells should be analysed, and also requests missing controls and clarifications.

In light of the reviews, we would like to invite you to revise the work to thoroughly address the reviewers' reports. Given the extent of revision needed, we cannot make a decision about publication until we have seen the revised manuscript and your response to the reviewers' comments. Your revised manuscript is likely to be sent for further evaluation by all or a subset of the reviewers.

In addition to these revisions, you will need to complete some formatting changes, which you will receive in a follow up email. A member of our team will be in touch with a set of requests shortly.

We expect to receive your revised manuscript within 3 months. Please email us (plosbiology@plos.org) if you have any questions or concerns, or would like to request an extension.

At this stage, your manuscript remains formally under active consideration at our journal; please notify us by email if you do not intend to submit a revision so that we may withdraw it.

**IMPORTANT - SUBMITTING YOUR REVISION**

Your revisions should address the specific points made by each reviewer. Please submit the following files along with your revised manuscript:

1. A 'Response to Reviewers' file - this should detail your responses to the editorial requests, present a point-by-point response to all of the reviewers' comments, and indicate the changes made to the manuscript.

*NOTE: In your point-by-point response to the reviewers, please provide the full context of each review. Do not selectively quote paragraphs or sentences to reply to. The entire set of reviewer comments should be present in full and each specific point should be responded to individually, point by point.

You should also cite any additional relevant literature that has been published since the original submission and mention any additional citations in your response.

2. In addition to a clean copy of the manuscript, please also upload a 'track-changes' version of your manuscript that specifies the edits made. This should be uploaded as a "Revised Article with Changes Highlighted" file type.

3. Resubmission Checklist

When you are ready to resubmit your revised manuscript, please refer to this resubmission checklist: https://plos.io/Biology_Checklist

To submit a revised version of your manuscript, please go to https://www.editorialmanager.com/pbiology/ and log in as an Author. Click the link labelled 'Submissions Needing Revision' where you will find your submission record.

Please make sure to read the following important policies and guidelines while preparing your revision and fulfil the editorial requests:

a) *PLOS Data Policy*

Please note that as a condition of publication PLOS' data policy (http://journals.plos.org/plosbiology/s/data-availability) requires that you make available all data used to draw the conclusions arrived at in your manuscript. If you have not already done so, you must include any data used in your manuscript either in appropriate repositories, within the body of the manuscript, or as supporting information (N.B. this includes any numerical values that were used to generate graphs, histograms etc.). Please also indicate in each figure legend where the data can be found. For an example see here: http://www.plosbiology.org/article/info%3Adoi%2F10.1371%2Fjournal.pbio.1001908#s5

b) *Published Peer Review*

Please note while forming your response, if your article is accepted, you may have the opportunity to make the peer review history publicly available. The record will include editor decision letters (with reviews) and your responses to reviewer comments. If eligible, we will contact you to opt in or out. Please see here for more details:

https://blogs.plos.org/plos/2019/05/plos-journals-now-open-for-published-peer-review/

c) *Blot and Gel Data Policy*

Please provide the original, uncropped and minimally adjusted images supporting all blot and gel results reported in an article's figures or Supporting Information files. We will require these files before a manuscript can be accepted so please prepare them now, if you have not already uploaded them. Please carefully read our guidelines for how to prepare and upload this data: https://journals.plos.org/plosbiology/s/figures#loc-blot-and-gel-reporting-requirements

d) *Blurb*

Please also provide a blurb which (if accepted) will be included in our weekly and monthly Electronic Table of Contents, sent out to readers of PLOS Biology, and may be used to promote your article in social media. The blurb should be about 30-40 words long and is subject to editorial changes. It should, without exaggeration, entice people to read your manuscript. It should not be redundant with the title and should not contain acronyms or abbreviations. For examples, view our author guidelines: https://journals.plos.org/plosbiology/s/revising-your-manuscript#loc-blurb

e) *Protocols deposition*

To enhance the reproducibility of your results, we recommend that if applicable you deposit your laboratory protocols in protocols.io, where a protocol can be assigned its own identifier (DOI) such that it can be cited independently in the future. For instructions see: https://journals.plos.org/plosbiology/s/submission-guidelines#loc-materials-and-methods

Thank you again for your submission to our journal. We hope that our editorial process has been constructive thus far, and we welcome your feedback at any time. Please don't hesitate to contact us if you have any questions or comments.

Sincerely,

Ines

--

Ines Alvarez-Garcia, PhD

Senior Editor

PLOS Biology

ialvarez-garcia@plos.org

------------------------------------

Reviewers' comments

Rev. 1:

This study identified a function for the C. elegans endonuclease LEM-3 in the separation of sister cells during the final cell division preceding terminal differentiation and fate determination. When LEM-3 is absent, chromatin bridges remain unresolved, leading to failed cytokinesis and the development of binucleate syncytia, evident by a "canal" remaining between sister cells. The failure to separate sister cells during cytokinesis impacts cell neuronal differentiation, resulting in sister cells in some cases acquiring the same cell fate. In some cases where the sibling cell fate is apoptosis, lem-3 mutants showed a loss of apoptosis. Studies of fluorescently labelled cell fate markers and cell fate determinants indicate that these proteins may diffuse in the canal. The results are mostly convincing, and the manuscript is well-written. However, although their study provides detailed explanations to the duplication of sister cell fate in cytokinesis mutants, one could argue that the findings are not surprising and do not in themselves point to a novel general principle in developmental biology.

Major issues

Figure 1: they refer to the connections between sister cell in lem3 mutants as "canals". But could instead be axon/dendrite projections between the cells. What is the difference between a "canal" and an aberrant axon/dendrite?

Figure 2: I find the labelling in the figure confusing. Are all these images from a lem-3 mutant background? How do they score that there is a syncytium? According to Figure 1, the "canal" is only formed in ~20% of cases.

Figure 3: The diffusion of proteins through the canal between the two sister cells is open for other interpretations. For instance, the presence of MEC3::GFP in both sister cells does not automatically mean that MEC3::GFP protein has diffused through the canal. Instead, the diffusion of other proteins may trigger the same cell fate in both sister cells and hence result in the expression of many cell fate markers. To definitively claim that MEC3::GFP and other proteins diffuse through the canal they would have to activate the reporter protein expressions at a later stage and study its diffusion.

Minor issues

Introduction, 1st paragraph: The "final round of cell division…" is over-simplified. In general terms, when introducing the topic and referring to many biological settings, it can come in several very different forms: 1) both sister cells differentiating, and into different fates, 2) both sister cells differentiating, but into the same fate, or 3) one cell differentiating, often referred to as the "daughter cell", while the other, the "mother cell" retains some level of progenitor properties and continues dividing.

Should not unk14 and unk85 be unc-14 and unc-85?

Rev. 2:

The manuscript by Deng and Zheng reports the characterization neuronal fate specification in animals lacking the function of LEM-3, a midbody-localized nuclease. The authors uncovered an allele of lem-3 in a screen for extra touch receptor neurons (TRNs). They show that these mutants maintain a stable cytoplasmic bridge (which they call intercellular canal) between TRNs and their sister cells. The bridge contains actin, tubulin and DNA/chromatin and enables cytoplasmic exchange between sister cells. The authors demonstrate that the bridge correlates with a proportion of sister cells adopting a TRN fate, likely due to the diffusion of TRN-promoting factors, including MEC-3. The authors propose that the presence of chromatin bridges, which are typically processed by LEM-3, results in the long-term stabilization of the intercellular bridge in terminally-differentiating neurons and that this impacts neuronal fates by enabling the exchange of proteins between the two sister cells.

Overall I find the manuscript to be of excellent quality, with a sound experimental design and original findings that should be of interest to neurobiologists and cell biologists alike. The main finding that chromatin bridges can result in the stabilization of the intercellular bridge in cells undergoing terminal division prior to differentiation is novel, and the authors convincingly show that this bridge enables the sharing of cytoplasmic constituents, and thus can have an impact on cell fate. I have a few suggestions that the authors should consider to improve the quality of their work.

My main suggestion is in regards to lines 133-149, where the authors postulate that the fate change in TRN sister cells is due to diffusion of the TRN fate specifying factor MEC-3. They seek to test this idea by showing that ectopic expression of MEC-3 is sufficient to induce a fate change, however I do not think that this ectopic expression really tests their idea. I understand that part of the idea is that molecules can diffuse through the stable canal, including MEC-3 itself (as shown in Figure 3), however this does not show that MEC-3 is functionally competent. I think that the simplest way to show that the fate change is due to the presence of MEC-3 is to test whether the fate change phenotype is suppressed by the loss of MEC-3. Of course such gross perturbation will not demonstrate that it is the MEC-3 present in the TNR cell that induces the fate change in its sister, however it should unambiguously assess whether the presence of MEC-3 is needed for this fate change. I think that this functional assessment is important given that the authors take for granted (in this and subsequent sections) that their fate change phenotype is caused by MEC-3 diffusion.

Minor suggestions and concerns

The authors refer to the connection as an intercellular canal, but this is a somewhat novel term that does not serve to anchor their work in biology. When cells divide they form a transient intercellular bridge around a midbody, and abscission leads to the severing of the bridge (membrane scission) and release of the midbody. In certain cells that undergo incomplete cytokinesis (e.g. germ cells), the intercellular bridge is stabilized, thus forming a stable intercellular bridge. I suggest that the authors employ the term stable intercellular bridge (or stable cytoplasmic bridge) instead of intercellular canal to better anchor their finding into the existing work on these structures.

I wonder whether the term syncytium is appropriate to refer to cells that remain connected by a stable bridge as a result of cytokinesis failure. My understanding of syncytia is that they can be generated by incomplete cytokinesis but this is a programed feature that is highly regulated, which is not the case here. They can also arise of pathological conditions (e.g. viral infections) but it is also different than what is observed here. I understand why the authors equate their cell pairs to syncytia but my opinion is that the term is not employed appropriately in this context. I suggest to refer to stably connected sister cells rather than syncytial cells.

Lines 60-61: As far as I know, whether the entire C. elegans germline is formed as a result of incomplete cytokinesis is still an open question. Previous work has shown that the germline founder cell undergoes incomplete cytokinesis (PMID: 29074566) and that the first division of the PGCs may be incomplete (PMID: 34195824), however the notion that there is a sustained incomplete cytokinesis program when the germline expands remains hypothetical and some work suggests other mechanisms. The authors could instead write that "the germline of C. elegans hermaphrodites arises via incomplete cytokinesis and forms a syncytium in which more than 1000 nuclei share cytoplasm through stable bridges".

Line 70: define TRN (touch receptor neuron) in this sentence.

Line 77, "…we mapped unk14 to the V618M mutation in lem-3…": Please rewrite. V618M is an amino acid change, not a mutation that can be mapped. The allele mapped to a change at the nucleotide level, which results in a V618M change in the resulting protein.

Lines 209-213 and Figure 3D: I find that the experiment to demonstrate that the stable canal is needed for cytoplasmic exchange is somewhat convoluted. The authors show that if they sever the canal they see a decrease in converted Dendra2 levels in the non-TNR sister, but this occurs over 48 hours. Have the authors considered performing the photoconversion in animals in which the canal has already been cut (i.e. essentially doing the same experiment as in 3B but after the canal is cut)? It seems like this would be more direct to demonstrate that the canal enables cytoplasmic exchange.

Lines 378-381 and Figure 6I: The authors report that inhibiting ced-3 (which blocks apoptosis) results in an increase in the surviving sister cell in cell pairs where one usually dies. I think that it would rather be more informative if the authors documented whether the decrease in 3-cell body syncytia that they reported in older animals (Figure 6E) is suppressed in ced-3 mutants. If this were the case it would support their claim that apoptosis is delayed in 3-cell syncytia.

Rev. 3:

Deng et al. utilize C. elegans as an experimental system to investigate how the failed cytokinesis affect the specification of postmitotic neuron cells. They found that the loss of LEM-3 endonuclease, which is responsible for chromatin bridges resolution during cytokinesis, leads to binucleate syncytia connecting TRNs and sister cells through intercellular canal. These intercellular canals allow the diffusion of cytoplasmic proteins, while the transcriptomes of the linked cells can be independently regulated. They further investigated the functional consequences of these connections and found that the cytoplasmic linkages between sister cells resulted in suppression of neuronal fates and prevention of apoptotic fates. Finally, they showed that LEM-3 has a general role in cell fate specification in the nervous system by resolving the chromatin bridges.

In general, this is a very exciting study that provides insight into the consequence of compromised cytokinesis in cell fate differentiation. The experiments are overall well executed, the results are clearly presented.

Major points:

1. While previous studies have shown LEM-3 recruitment to the midbody in embryos, its localization should also be examined in the division that produces TRNs and their sister cells.

2. Fig 1E. The authors should discuss the potential reasons for the significant variation in syncytium penetrance among different TRN subtypes.

3. Fig. 3 shows that proteins, but not mRNAs, diffuse freely throughout the syncytium. Could it be that mRNAs are degraded in the canal or actively blocked by a selective barrier?

4. Fig. 4A The analysis should be complemented by a control experiment demonstrating syncytium formation in wild-type worms treated with the DNA topoisomerase II inhibitor or subjected to mcm-7 RNAi.

5. Fig 6E shows that although the three cells are connected by intercellular canals, only the third cell can be removed without triggering programmed cell death in the entire syncytium. Given that cytoplasmic proteins can diffuse throughout the syncytium, it would be interesting to investigate whether the other two cells could also be labeled by an apoptotic marker such as CED-1::GFP.

Minor points:

1. The abbreviation "TRN" should be spelled out as "touch receptor neuron (TRN)" upon its first use in the manuscript.

2.line 122 Fig. 1B does not seem relevant to this specific point. The supporting data appears to be in Fig.S2 A-B only.

3. Please indicate how the "~20%" value was quantified and where the data is presented. It is not evident in the current Fig. 4E. Also, statistical analysis is required for the quantitative data in Fig 4E.

4. Please add the figure citation (Fig. 5D) to the sentence on lines 307-311 regarding drug treatments.

# Author comment: Author response to Decision Letter 2

**DOI:** 10.1371/journal.pbio.3004099.r003

25 Sep 2026

Attachment Submitted filename: Response to Reviewer Feedback for PBIOLOGY.docx

# Peer review: Decision Letter 2

**DOI:** 10.1371/journal.pbio.3004099.r004

**Authors:** Ines Alvarez-Garcia

9 Oct 2026

Dear Dr Zheng,

Thank you for your patience while we considered your revised manuscript entitled "LEM-3/ANKLE1 nuclease prevents the formation of abnormal syncytium between postmitotic sister cells and safeguards neuronal differentiation" for publication as a Research Article at PLOS Biology. This revised version of your manuscript has been evaluated by the PLOS Biology editors, the Academic Editor and two of the original reviewers.

Based on the reviews (attached below), we are likely to accept this manuscript for publication, provided you satisfactorily address the data and other policy-related requests stated below my signature.

In addition, we would like you to consider a suggestion to improve the title:

"LEM-3/ANKLE1 nuclease prevents the formation of abnormal syncytium to safeguard neuronal differentiation"

As you address these items, please take this last chance to review your reference list to ensure that it is complete and correct. If you have cited papers that have been retracted, please include the rationale for doing so in the manuscript text, or remove these references and replace them with relevant current references. Any changes to the reference list should be mentioned in the cover letter that accompanies your revised manuscript.

In addition to these revisions, you may need to complete some formatting changes, which you will receive in a follow up email. A member of our team will be in touch with a set of requests shortly. If you do not receive a separate email within a few days, please assume that checks have been completed, and no additional changes are required.

We expect to receive your revised manuscript within two weeks.

To submit your revision, please go to https://www.editorialmanager.com/pbiology/ and log in as an Author. Click the link labelled 'Submissions Needing Revision' to find your submission record. Your revised submission must include the following:

- a cover letter that should detail your responses to any editorial requests, if applicable, and whether changes have been made to the reference list

- a Response to Reviewers file that provides a detailed response to the reviewers' comments (if applicable, if not applicable please do not delete your existing 'Response to Reviewers' file.)

- a track-changes file indicating any changes that you have made to the manuscript.

NOTE: If Supporting Information files are included with your article, note that these are not copyedited and will be published as they are submitted. Please ensure that these files are legible and of high quality (at least 300 dpi) in an easily accessible file format. For this reason, please be aware that any references listed in an SI file will not be indexed. For more information, see our Supporting Information guidelines:

https://journals.plos.org/plosbiology/s/supporting-information

*Published Peer Review History*

Please note that you may have the opportunity to make the peer review history publicly available. The record will include editor decision letters (with reviews) and your responses to reviewer comments. If eligible, we will contact you to opt in or out. Please see here for more details:

https://plos.org/published-peer-review-history/

*Press*

Should you, your institution's press office or the journal office choose to press release your paper, please ensure you have opted out of Early Article Posting on the submission form. We ask that you notify us as soon as possible if you or your institution is planning to press release the article.

*Protocols deposition*

To enhance the reproducibility of your results, we recommend that if applicable you deposit your laboratory protocols in protocols.io, where a protocol can be assigned its own identifier (DOI) such that it can be cited independently in the future. Additionally, PLOS ONE offers an option for publishing peer-reviewed Lab Protocol articles, which describe protocols hosted on protocols.io. Read more information on sharing protocols at https://plos.org/protocols?utm_medium=editorial-email&utm_source=authorletters&utm_campaign=protocols

Please do not hesitate to contact me should you have any questions.

Sincerely,

Ines

--

Ines Alvarez-Garcia, PhD

Senior Editor

PLOS Biology

ialvarez-garcia@plos.org

------------------------------------------------------------------------

DATA POLICY: IMPORTANT - PLEASE READ

You may be aware of the PLOS Data Policy, which requires that all data be made available without restriction: http://journals.plos.org/plosbiology/s/data-availability. For more information, please also see this editorial: http://dx.doi.org/10.1371/journal.pbio.1001797

Note that we do not require all raw data. Rather, we ask that all individual quantitative observations that underlie the data summarized in the figures and results of your paper be made available in one of the following forms:

1) Supplementary files (e.g., excel). Please ensure that all data files are uploaded as 'Supporting Information' and are invariably referred to (in the manuscript, figure legends, and the Description field when uploading your files) using the following format verbatim: S1 Data, S2 Data, etc. Multiple panels of a single or even several figures can be included as multiple sheets in one excel file that is saved using exactly the following convention: S1_Data.xlsx (using an underscore).

2) Deposition in a publicly available repository. Please also provide the accession code or a reviewer link so that we may view your data before publication.

Regardless of the method selected, please ensure that you provide the individual numerical values that underlie the summary data displayed in the following figure panels as they are essential for readers to assess your analysis and to reproduce it:

Fig. 1E; Fig. 3C, E; Fig. 4A, B, E, F, G, H; Fig. 5E, F; Fig. 6E, H, I; Fig. 7F, I; Fig. S1B; Fig. S7B, D, F and Fig. S8A

NOTE: the numerical data provided should include all replicates AND the way in which the plotted mean and errors were derived (it should not present only the mean/average values).

Please also ensure that figure legends in your manuscript include information on WHERE THE UNDERLYING DATA CAN BE FOUND, and ensure your supplemental data file/s has a legend.

Please ensure that your Data Statement in the submission system accurately describes where your data can be found.

------------------------------------------------------------------------

CODE POLICY

Per journal policy, if you have generated any custom code during the course of this investigation, please make it available without restrictions. Please ensure that the code is sufficiently well documented and reusable, and that your Data Statement in the Editorial Manager submission system accurately describes where your code can be found. More information on our Code Policy, what and how to share can be found here: https://journals.plos.org/plosbiology/s/code-availability

Please note that we cannot accept sole deposition of code in GitHub, as this could be changed after publication. However, you can archive this version of your publicly available GitHub code to Zenodo. Once you do this, it will generate a DOI number, which you will need to provide in the Data Accessibility Statement (you are welcome to also provide the GitHub access information). See the process for doing this here: https://docs.github.com/en/repositories/archiving-a-github-repository/referencing-and-citing-content

------------------------------------------------------------------------

Reviewers' comments

Rev. 2:

I consider the revised manuscript by Deng and Zheng as significantly improved compared to the previous version. The authors have done an excellent job to address all reviewers' comments, both editorially and experimentally. The work is of excellent quality and it makes a significant contribution to our understanding of terminal neuronal cell differentiation during development, particularly in the context of cytokinesis failure induced by chromatin bridges.

Rev. 3: Ye Hong - please note that this reviewer has signed the review.

The authors have addressed most of my concerns satisfactorily. Additional experiments and data largely support their conclusions. Regarding my point 3, the authors did not directly address whether mRNAs are degraded in the canal or actively blocked by a selective barrier, but this is a technically challenging question and remains open for future investigation. Overall, all other points have been adequately addressed and the revised manuscript has been improved significantly. I recommend acceptance of this manuscript for publication.

# Author comment: Author response to Decision Letter 3

**DOI:** 10.1371/journal.pbio.3004099.r005

10 Oct 2026

Attachment Submitted filename: Response_to_Reviewer_Feedback_for_PBIOLOGY_auresp_3.docx

# Editor report: Decision Letter 3

**DOI:** 10.1371/journal.pbio.3004099.r006

**Authors:** Ines Alvarez-Garcia

23 Oct 2026

Dear Dr Zheng,

Thank you for the submission of your revised Research Article entitled "LEM-3/ANKLE1 nuclease prevents the formation of abnormal syncytium to safeguard neuronal differentiation" for publication in PLOS Biology. On behalf of my colleagues and the Academic Editor, François Schweisguth, I am delighted to let you know that we can in principle accept your manuscript for publication, provided you address any remaining formatting and reporting issues. These will be detailed in an email you should receive within 2-3 business days from our colleagues in the journal operations team; no action is required from you until then. Please note that we will not be able to formally accept your manuscript and schedule it for publication until you have completed any requested changes.

Please take a minute to log into Editorial Manager at http://www.editorialmanager.com/pbiology/, click the "Update My Information" link at the top of the page, and update your user information to ensure an efficient production process.

PRESS

We frequently collaborate with press offices. If your institution or institutions have a press office, please notify them about your upcoming paper at this point, to enable them to help maximise its impact. If the press office is planning to promote your findings, we would be grateful if they could coordinate with biologypress@plos.org. If you have previously opted in to the early version process, we ask that you notify us immediately of any press plans so that we may opt out on your behalf.

We also ask that you take this opportunity to read our Embargo Policy regarding the discussion, promotion and media coverage of work that is yet to be published by PLOS. As your manuscript is not yet published, it is bound by the conditions of our Embargo Policy. Please be aware that this policy is in place both to ensure that any press coverage of your article is fully substantiated and to provide a direct link between such coverage and the published work. For full details of our Embargo Policy, please visit http://www.plos.org/about/media-inquiries/embargo-policy/.

Many congratulations and thanks again for choosing PLOS Biology for publication and supporting Open Access publishing. We look forward to publishing your study.

Sincerely,

Ines

--

Ines Alvarez-Garcia, PhD

Senior Editor

PLOS Biology

ialvarez-garcia@plos.org
